# 14-3-3ε Overexpression Contributes to Epithelial-Mesenchymal Transition of Hepatocellular Carcinoma

**DOI:** 10.1371/journal.pone.0057968

**Published:** 2013-03-06

**Authors:** Tzu-An Liu, Yee-Jee Jan, Bor-Sheng Ko, Shu-Man Liang, Shyh-Chang Chen, John Wang, Chiun Hsu, Yao-Ming Wu, Jun-Yang Liou

**Affiliations:** 1 Institute of Cellular and System Medicine, National Health Research Institutes, Zhunan, Taiwan; 2 Department of Pathology and Laboratory Medicine, Taichung Veterans General Hospital, Taichung, Taiwan; 3 Department of Internal Medicine, National Taiwan University Hospital, Taipei, Taiwan; 4 Department of Oncology, National Taiwan University Hospital, Taipei, Taiwan; 5 Department of Surgery, National Taiwan University Hospital, Taipei, Taiwan; 6 Graduate Institute of Basic Medical Science, China Medical University, Taichung, Taiwan; Seoul National University, Republic of Korea

## Abstract

**Background:**

14-3-3ε is implicated in regulating tumor progression, including hepatocellular carcinoma (HCC). Our earlier study indicated that elevated 14-3-3ε expression is significantly associated with higher risk of metastasis and lower survival rates of HCC patients. However, the molecular mechanisms of how 14-3-3ε regulates HCC tumor metastasis are still unclear.

**Methodology and Principal Findings:**

In this study, we show that increased 14-3-3ε expression induces HCC cell migration and promotes epithelial-mesenchymal transition (EMT), which is determined by the reduction of E-cadherin expression and induction of N-cadherin and vimentin expression. Knockdown with specific siRNA abolished 14-3-3ε-induced cell migration and EMT. Furthermore, 14-3-3ε selectively induced Zeb-1 and Snail expression, and 14-3-3ε-induced cell migration was abrogated by Zeb-1 or Snail siRNA. In addition, the effect of 14-3-3ε-reduced E-cadherin was specifically restored by Zeb-1 siRNA. Positive 14-3-3ε expression was significantly correlated with negative E-cadherin expression, as determined by immunohistochemistry analysis in HCC tumors. Analysis of 14-3-3ε/E-cadherin expression associated with clinicopathological characteristics revealed that the combination of positive 14-3-3ε and negative E-cadherin expression is significantly correlated with higher incidence of HCC metastasis and poor 5-year overall survival. In contrast, patients with positive 14-3-3ε and positive E-cadherin expression had better prognostic outcomes than did those with negative E-cadherin expression.

**Significance:**

Our findings show for the first time that E-cadherin is one of the downstream targets of 14-3-3ε in modulating HCC tumor progression. Thus, 14-3-3ε may act as an important regulator in modulating tumor metastasis by promoting EMT as well as cell migration, and it may serve as a novel prognostic biomarker or therapeutic target for HCC.

## Introduction

Epithelial-mesenchymal transition (EMT), a developmental process by which epithelial cells reduce cell-cell adhesion and lose apical-basal cell polarity, plays a critical role in the embryogenesis and conversion of early stage tumors into aggressive malignancies [1]–[3]. EMT promotes multiple physiological processes that increase the invasiveness and metastasis potentials of human tumors [1]–[3]. EMT is typically characterized by loss of E-cadherin, gain of N-cadherin and vimentin, and translocation of β-catenin from membrane to the nuclear compartment [4], [5]. The impairment of E-cadherin is a hallmark of EMT, and E-cadherin expression is often inversely correlated with tumor malignancy and patient survival [4], [5]. The E-cadherin expression level is down-regulated by gene silencing with CpG methylation on promoter in hepatocellular carcinoma (HCC) [6], [7] and may be associated with tumor grade and poor prognosis of HCC [8]. Several transcriptional regulators that act as E-cadherin repressors are mediated by recognizing the E-box motif on the E-cadherin promoter region [9]–[11]. Factors of Snail zinc finger, Zeb and bHLH families are known to suppress E-cadherin, thereby promoting the EMT process and tumor metastasis [9]–[11]. In addition, increased Zeb-1, Snail, SIP1, and Twist expressions are reportedly associated with the clinicopathological significances of HCC malignant progression, including cancer invasion and poor patient survival [12]–[16].

14-3-3 proteins are a family of regulatory molecules with highly conserved homology among all eukaryotic cells [17], [18]. 14-3-3 modulates physiological functions *via* binding intracellular proteins with Ser/Thr phosphorylation-dependent domains, thereby influencing conformation, activity, subcellular localization, and protein complex stability [17], [18]. 14-3-3 proteins comprise seven isoforms (β, ε, γ, η, σ, τ/θ and ζ), which play crucial roles in regulating multiple cellular processes, including cell cycle regulation, DNA repair, apoptosis, cell adhesion, and motility [17], [18]. 14-3-3 proteins have been implicated in various types of human malignancies [19]–[26]. Among the variety of 14-3-3 isoforms, increased expression of 14-3-3ε has been demonstrated in breast cancer, lung cancer, vulvar squamous cell carcinoma, follicular and papillary thyroid tumors, meningioma, and HCC [21], [24], [27]–[30], although reduced expression of 14-3-3ε in gastric cancer has been reported [31]. In addition, previous studies have demonstrated that up-regulation of 14-3-3ε expression protects colorectal cancer cells and endothelial cells from oxidative-stress induced apoptosis [32], [33], while suppression of 14-3-3ε by nonsteroidal anti-inflammatory drugs induces cancer and endothelial cell death [33], [34]. Furthermore, elevated expression of 14-3-3ε is significantly associated with increased metastatic risk, shortened overall survival, and progression-free survival of HCC [30]. Enhanced expression of 14-3-3ε was suggested to induce and be associated with focal adhesion kinase (FAK) expression *via* activation of NFκB signaling [35]. These results implied that 14-3-3ε was involved in the modulation of cell polarization and migration, which may potentially regulate HCC tumor development and metastasis. In this study, we show for the first time that 14-3-3ε induces HCC cell migration and EMT *via* regulation of Zeb-1/E-cadherin expression. Our results reveal that E-cadherin is a downstream modulator for 14-3-3ε during HCC tumor progression.

## Materials and Methods

### Cell Culture and Stable Cells

Huh-7 (Japanese Collection of Research Bioresources, JCRB-0403), HepG2 (American Type Culture Collection, ATCC-HB-8065), Hep3B (ATCC-HB-8064), PLC-5 (ATCC-CRL-8024) and SK-Hep1 (ATCC-HTB-52) human hepatocellular carcinoma cells were maintained in DMEM (Gibco, Gaithersburg, MD) supplemented with 10% fetal bovine serum (FBS; Hyclone Thermo Fisher Scientific, Waltham, MA), 100 units/ml penicillin, and 100 units/ml streptomycin, in a humidified incubator (Forma) with 5% CO_2_ at 37°C. For stable transfection, 14-3-3ε cDNA was amplified by PCR and then subcloned into the p3XFlag-CMV vector. Huh-7 cells were transfected with p3XFlag-CMV (Control) or p3XFlag-14-3-3ε (14-3-3ε) by use of Polyjet™ transfection reagent (SignaGen Laboratories, Rockville, MD) according to the manufacturer’s instructions. The transfected cells were selected with G418 (500 µg/ml) for 4 weeks. Single colonies of stable clones (at least 3 in each cell line) were maintained in DMEM with 10% FBS and 200 µg/ml of G418.

### Transient Transfection

Huh-7 and HepG2 cells were transiently transfected with control and 14-3-3ε by use of Polyjet™ transfection reagent (Signa-Gen Laboratories, Ijamsville, MD). Cells were transfected with control or 0.5 to 1.5 µg of 14-3-3ε vectors per 6-well plate followed by incubation with Polyjet™/DNA complex-containing medium and replaced with complete medium for 24 hours. Transfected cells were incubated for additional 24 hours before performing cell migration assays or protein expression analysis.

### Knockdown Studies

Gene silencing was performed using 14-3-3ε, Snail (Stealth RNAi, Invitrogen, Carlsbad, CA), Zeb-1 siRNAs (Santa Cruz, Heidelberg, Germany) and Stealth RNA Negative Control (Invitrogen, Carlsbad, CA) with reported sequences (Table S1). Transient transfection of siRNA was carried out using Lipofectamine™ RNAiMAX (Invitrogen, Grand Island, NY) according to the manufacturer’s guidelines.

### Western Blot Analysis

Cells were lysed in ice-cold RIPA buffer (0.5 M Tris-HCl, pH 7.4, 1.5 M NaCl, 2.5% deoxycholic acid, 10% NP-40, 10 mM EDTA, Millipore, Temecula, CA) containing cocktail protease inhibitors (Roche, IN, USA). Cell lysates were centrifuged at 15,000 rpm for 20 minutes at 4°C, and protein concentrations were determined by a Bio-Rad protein assay kit (Bio-Rad Laboratories, Hercules, CA). Each sample of 20 µg protein was applied to a gradient SDS-PAGE gel and immunoblotted onto PVDF membranes. The membranes were blocked and probed with indicated primary antibodies of Flag and actin (Sigma-Aldrich, St. Louis, MO), E-cadherin and N-cadherin (BD Biosciences, San Jose, CA), 14-3-3ε, Zeb-1, Twist and Slug (Santa Cruz Biotechnologies, Heidelberg, Germany), Snail (Cell Signaling Technology, Beverly, MA), Zeb-2 (Abcam PLC, Cambridge, UK), and vimentin (Millipore, Temecula, CA). The membranes were immersed in PBST containing horseradish peroxidase-conjugated secondary antibody, and protein levels were determined by use of enhanced chemiluminescence reagents.

### Immunofluorescence Staining

Immunofluorescence staining was performed as described previously [25]. Briefly, 14-3-3ε and control cells were fixed with 2% paraformaldehyde for 15 minutes at 4°C. After washing, cells were permeabilized with 0.1% Triton X-100 in PBS for 5 minutes and blocked with PBS containing 10% FBS at room temperature for 1 hour. For the immunofluorescence staining, cells were incubated with the primary antibodies of anti-E-cadherin and anti-N-cadherin (BD Biosciences, San Jose, CA), and anti-vimentin (Millipore, Temecula, CA) in PBS containing 1% FBS at 4°C overnight, followed by incubation with Alexa Fluor® 488 secondary antibody (Invitrogen, Grand Island, NY) in PBS containing 5% bovine serum albumin at room temperature for 2 hours. Samples were mounted and images were analyzed by use of the Leica TCS SP5 Confocal Imaging System (Leica, Germany).

### Migration Assay

Bio-coat cell migration Boyden chambers were used for cell migration assay (Becton Dickinson, Pont-de-Claix, France). Briefly, cells were trypsinized and suspended in 0.1% BSA-DMEM and cells (1×10^4^ for SK-Hep1, 6×10^4^ for Huh-7 and 2×10^5^ for HepG2) were added to the upper wells with 8-µm pores. Cells were allowed to migrate toward the bottom wells containing 100 µg/ml fibronectin (Becton Dickinson, Pont-de-Claix, France), epithelial growth factor (EGF, 20 ng/ml, Sigma-Aldrich, St. Louis, MO) and 10% BSA-DMEM for 20 hours. Cells remaining on the upper side were removed, and migrated cells on the bottom side were fixed and stained with 0.1% crystal violet containing 20% ethanol and 1% formadehyde for 20 minutes. Cell migration was quantified by counting the total number of migrated cells.

### Quantitative Real-time PCR

As described previously [25], [36], total RNA was extracted by use of the RNAspin Mini Kit (GE Healthcare, Freiburg, Germany). cDNA was synthesized from 2–5 µg RNA by use of the oligo(dT)_18_ primers and RevertAid™ First Strand cDNA Synthesis Kit (Fermentas, Thermo Fisher Scientific, Waltham, MA). Quantitative real-time PCR involved use of SYBR Green (Kapa biosystem, Woburn, MA) with specific oligonucleotide primers (Table S2) from the AB 7900HT system (Applied Biosystems, USA). Applied Biosystems Relative Quantification (RQ) Manager Software version 1.2 was used to analyze the relative gene expression in each sample by the comparative Ct method. Gene expression was normalized to that of glyceraldehyde-3-phosphate dehydrogenase (GAPDH).

### Clinical Specimens

Tissue samples were obtained from 113 HCC patients who had undergone surgery for tumor resection or biopsy at Taichung Veterans General Hospital from January 1999 to December 2001. The mean follow-up time was 51.5±28.7 months. Thirty-three patients (29.2%) developed tissue-proved metastasis in 3 to 87 months after the resection of primary HCC. Slides from paraffin-embedded surgical specimens of primary tumors with surrounding non-cancerous liver parenchyma were subjected to immunohistochemical (IHC) staining. The pathological features, IHC staining results, clinical parameters, including Barcelona-Clinic Liver Cancer (BCLC) staging [37], and disease outcomes were collected for analysis. This study was approved by the Institutional Review Board of Taichung Veterans General Hospital. The policy that no informed consents are required for using these de-linked samples for retrospective analysis was also approved by the Institutional Review Board.

### Immunohistochemical Analysis

For immunohistochemistry analysis, an automatic immunostaining device and ultraView detection kit (Ventana XT Medical System, Tucson, AZ) were used to detect 14-3-3ε expression in paraffin-embedded tissues by use of a primary antibody against 14-3-3ε (1∶800; Santa Cruz Biotechnology, Santa Cruz, CA) and E-cadherin (1∶800; BD Bioscience). A negative control was prepared by the same staining procedure without primary antibodies. The intensity of 14-3-3ε and E-cadherin protein staining was semiquantitatively scored by a Quick-score (Q-score) method based on intensity and heterogeneity [28]–[30], [38]–[40]. Staining intensity was scored as 0 (negative), 1 (weak), 2 (moderate), or 3 (strong). For heterogeneity, the proportions of tumor cells positively stained with 14-3-3ε and E-cadherin were scored as 0 (0%); 1 (1–25%); 2 (26–50%); 3 (51–75%) or 4 (76–100%). The Q-score of a given tissue sample was the sum of the intensity and heterogeneity scores and ranged from 0 to 7. A Q-score ≥2 was considered overexpressed, or positive expression, and a Q-score <2 was considered normal, or negative expression. Cases with <5% weakly stained specimens were considered as negative expression.

### Statistical Analysis

The Student’s *t*-test was used to analyze differences between 2 groups. Kaplan-Meier curves were plotted and the log rank test was used to analyze time-related variables of probabilities for metastasis and overall survival. A *P* value <0.05 was considered statistically significant.

## Results

### 14-3-3ε Promotes HCC Cell Migration

**Figure 1:**
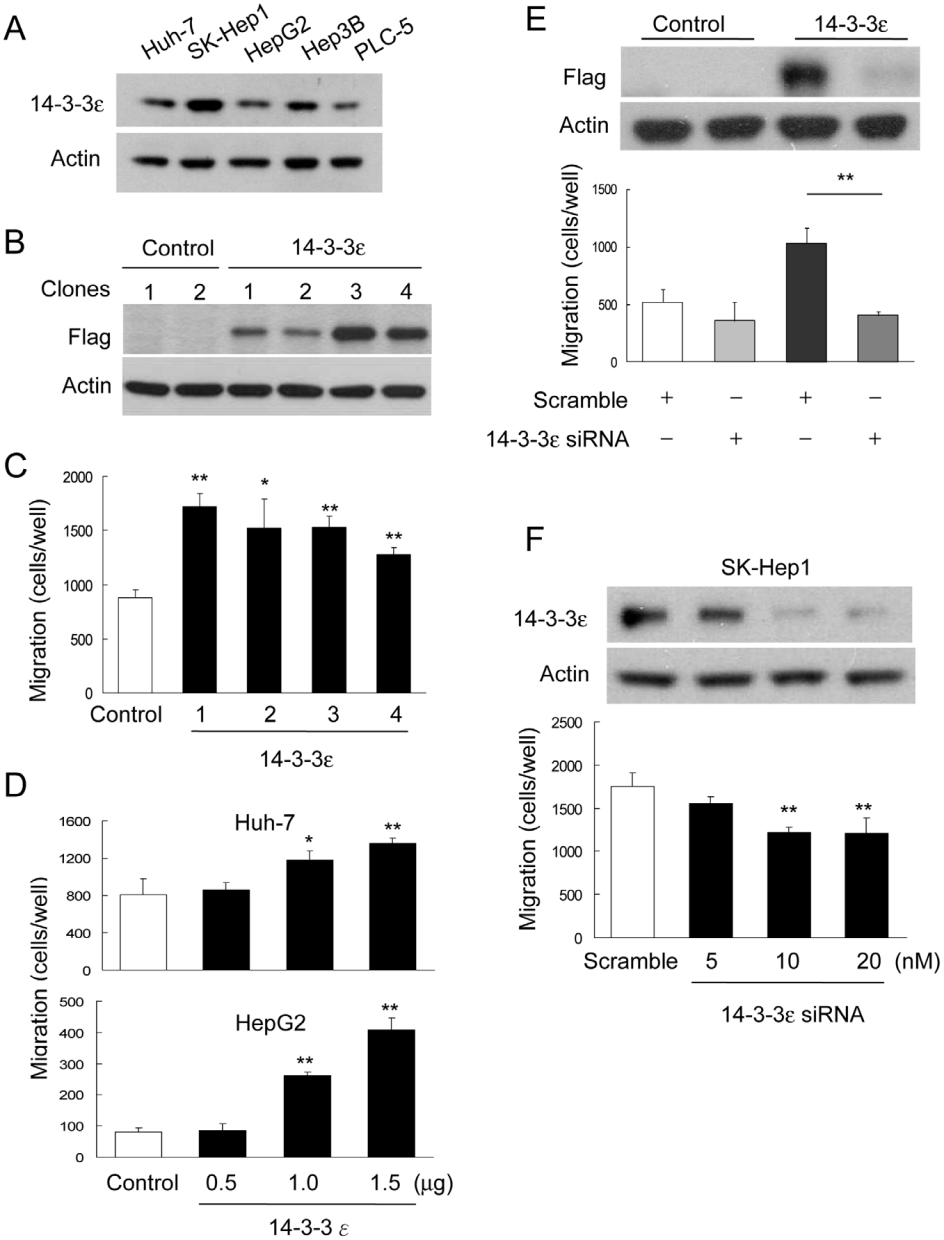
14-3-3ε induces cell migration. (A) Endogenous level of 14-3-3ε was determined in HCC cell lines including Huh-7, SK-Hep1, HepG2, Hep3B and PLC-5 cells by Western blotting analysis. (B) 14-3-3ε overexpression stable cells were confirmed by Western blotting analysis. Control, stable clones of empty vector (p3XFlag); 14-3-3ε, stable clones of p3XFlag-14-3-3ε. (C) Cell migratory abilities of 14-3-3ε overexpression and control cells were determined by Boyden chamber assay. (D) Effect of cell migration by transient and dose-dependent transfection of 14-3-3ε in Huh-7 and HepG2 cells. (E) Cells transfected with scramble or 14-3-3ε siRNA were subjected to detect the reduction of 14-3-3ε expression (upper panel) by Western blotting and cell migration (lower panel). Knockdown of 14-3-3ε with siRNA significantly inhibited 14-3-3ε-induced cell migration. (F) Knockdown of 14-3-3ε inhibited cell migration with a dose-dependently manner in SK-Hep1 cells. These data are from three independent experiments. Scale bars: mean ± SD. *P<0.05, **P<0.01.

To explore the potential role of 14-3-3ε in HCC tumor metastasis, we examined the expression of 14-3-3ε in distinct HCC cell lines. 14-3-3ε was detected in all tested HCC cells (Figure 1A). The well-differentiated HCC cells, including Huh-7, HepG2 and PLC-5, expressed lower levels of 14-3-3ε, while the poorly differentiated SK-Hep1 cells expressed higher levels (Figure 1A). We next established a stable cell line with 14-3-3ε overexpression. Huh-7 cells were transfected with p3XFlag-CMV (Control) or p3XFlag-14-3-3ε (14-3-3ε) vectors and selected by G418 for 4 weeks. Individual colonies were picked and 14-3-3ε expression was confirmed by Western blot analysis (Figure 1B). At least 3 clones were selected, and the representative clone was used for further experiments. To investigate whether 14-3-3ε regulates cell migration, we performed the migration assay with Boyden chamber experiments. We found that 14-3-3ε (stable clones 1–4) significantly induced cell migration (Figure 1C). In addition, the induction of cell migration accessed by 14-3-3ε overexpression was confirmed by transient transfection in both of Huh-7 and HepG2 cells. Transiently 14-3-3ε overexpression dose-dependently increased Huh-7 and HepG2 cell migration (Figure 1D). To further confirm the effect of 14-3-3ε on modulating cell migration, control or 14-3-3ε stable cells (clone 1) were transfected with scramble or 14-3-3ε siRNAs and the efficiency of 14-3-3ε knockdown was determined by Western blotting analysis (Figure 1E, upper panel). Knockdown with siRNA significantly abolished 14-3-3ε-induced cell migration (Figure 1E, lower panel). Additionally, knockdown of 14-3-3ε with siRNA significantly suppressed SK-Hep1 cell migration with a dose-dependent manner (Figure 1F). These results suggest that 14-3-3ε plays an important role in promoting HCC cell migration.

### 14-3-3ε Promotes Epithelial-mesenchymal Transition of HCC

**Figure 2:**
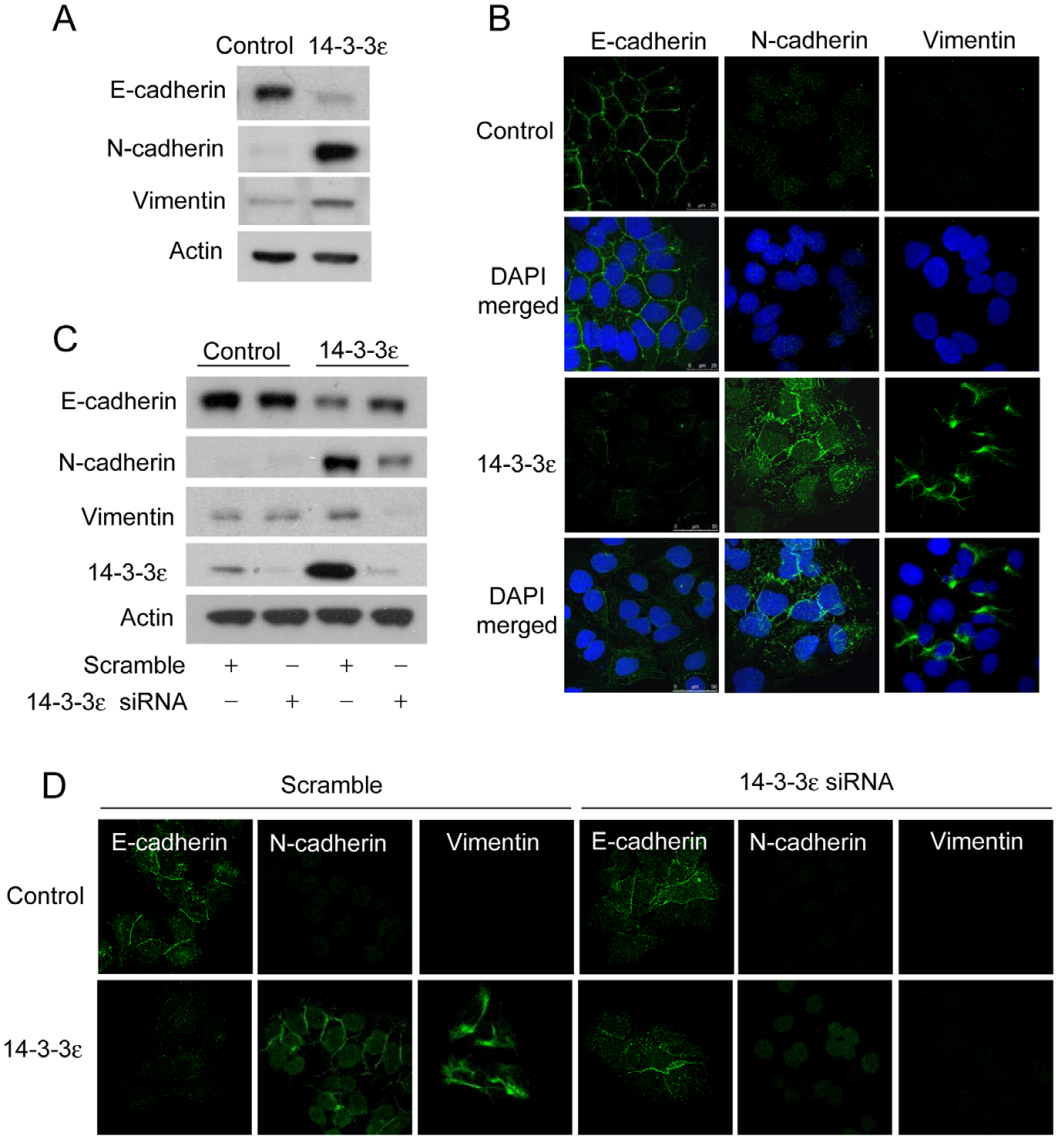
14-3-3ε promotes epithelial-mesenchymal transition. (A) Expressions of epithelial and mesenchymal markers included E-cadherin, N-cadherin, and vimentin in control and 14-3-3ε overexpression cells were determined by Western blot analysis and by (B) Immunofluorescent confocal microscopy. (C) 14-3-3ε siRNA-restored E-cadherin and -reduced N-cadherin as well as vimentin expression were determined by Western blotting analysis and by (D) Immunofluorescent confocal microscopy. Actin was used as loading control for protein determination.

To investigate whether 14-3-3ε expression regulates EMT of HCC cells, we determined the expression of EMT markers, E-cadherin, N-cadherin and vimentin, by Western blotting analysis. We found that 14-3-3ε overexpression significantly reduced E-cadherin expression, but it induced N-cadherin and vimentin expression (Figure 2A). The expression levels and subcellular localizations of E-cadherin, N-cadherin, and vimentin were further examined by immunofluorescent confocal microscopy. E-cadherin expression was detected at the cell-cell contacts in control cells, while it was dramatically reduced in 14-3-3ε overexpression cells (Figure 2B, left panel). Slight expression of N-cadherin and vimentin was detected in control cells, but such expression was significantly induced by 14-3-3ε overexpression (Figure 2B, middle and right panels). Furthermore, reduction of E-cadherin expression and the induction of N-cadherin and vimentin expression in 14-3-3ε overexpression cells were abrogated by transfection with 14-3-3ε siRNA, as determined by Western blotting analysis (Figure 2C). The regulation of these expressions of E-cadherin, N-cadherin and vimentin by 14-3-3ε knockdown were further confirmed by confocal microscopy. 14-3-3ε siRNA restored E-cadherin expression, which localized at the cell junctions (Figure 2D). In addition, knockdown with siRNA suppressed the N-cadherin and vimentin expression induced by 14-3-3ε (Figure 2D). These results indicate that 14-3-3ε overexpression promotes EMT of HCC.

### 14-3-3ε Promotes HCC Cell Migration via Upregulation of Zeb-1 and Snail

**Figure 3:**
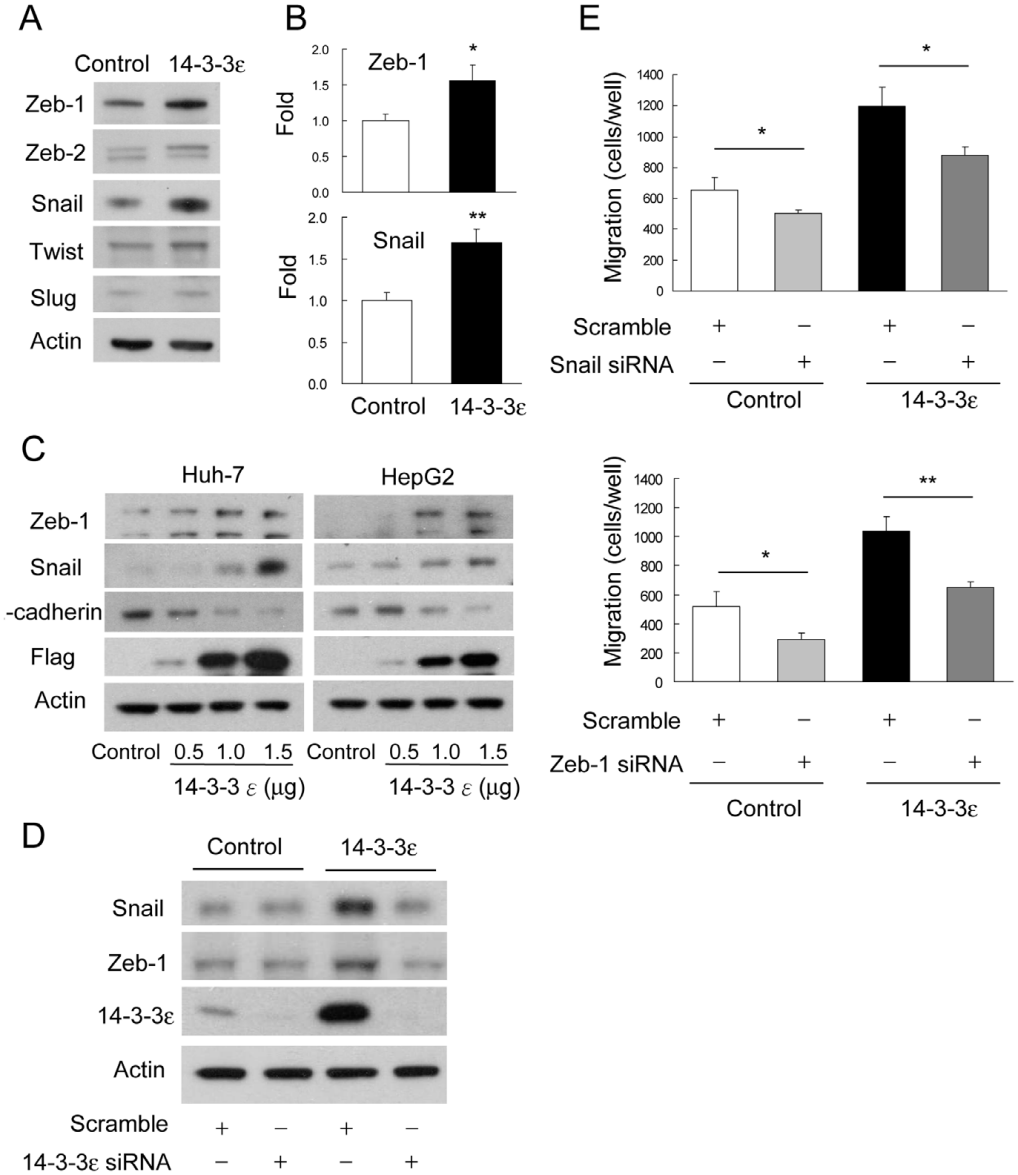
14-3-3ε induces Zeb-1 and Snail expression. (A) Western blotting analysis of Zeb-1, Zeb-2, Snail, Twist, and Slug expression in control and 14-3-3ε overexpression cells. Actin was used as loading control. (B) Quantitative real-time PCR analysis of Zeb-1 and Snail expression in control and 14-3-3ε overexpression cells. Scale bars: mean ± SD. *P<0.05, **P<0.01. (C) Effects of Zeb-1, Snail and E-cadherin expression by transient and dose-dependent transfection of 14-3-3 were analyzed by Western blotting analysis. Actin was used as loading control. (D) 14-3-3ε-induced Snail and Zeb-1 expression was suppressed by 14-3-3ε siRNA knockdown compared with scramble siRNA. Actin was used as loading control. (E) Cells were transfected with scramble, Snail or Zeb-1 siRNAs for 48 hours and cell migration was determined by Boyden chamber assay. 14-3-3ε–induced cell migration was abrogated by Snail or Zeb-1 siRNA knockdown. These data are from three independent experiments. Scale bars: mean ± SD. *P<0.05, **P<0.01.

To understand the molecular regulation of how 14-3-3ε induces EMT and reduces E-cadherin expression in HCC, we examined the expression levels of distinct E-box transcriptional suppressors. We found that 14-3-3ε overexpression selectively induced Zeb-1 and Snail expression but had no significant effect on Zeb-2, Twist or Slug (Figure 3A). The induced expression of Zeb-1 and Snail by 14-3-3ε was further confirmed by quantitative real-time PCR analysis (Figure 3B). In addition, results of transient transfection indicated that overexpression of 14-3-3ε dose-dependently induced Zeb-1/Snail and reduced E-cadherin expression in Huh-7 (Figure 3C, left panel) and HepG2 cells (Figure 3C, right panel). Furthermore, 14-3-3ε-induced expression of Zeb-1 and Snail was abrogated by knockdown of 14-3-3ε with siRNA (Figure 3D). These findings suggest that Zeb-1 and Snail may be involved in 14-3-3ε-induced HCC cell migration and EMT. We next determined the role of 14-3-3ε-induced Zeb-1 and Snail on cell migration. We found that knockdown of either Zeb-1 or Snail expression by siRNA significantly abolished 14-3-3ε induced cell migration (Figure 3E). These results indicate that Zeb-1 and Snail play important roles in regulating 14-3-3ε-induced HCC cell migration.

### 14-3-3ε Suppresses E-cadherin Expression Selectively Mediated by Induction of Zeb-1

**Figure 4:**
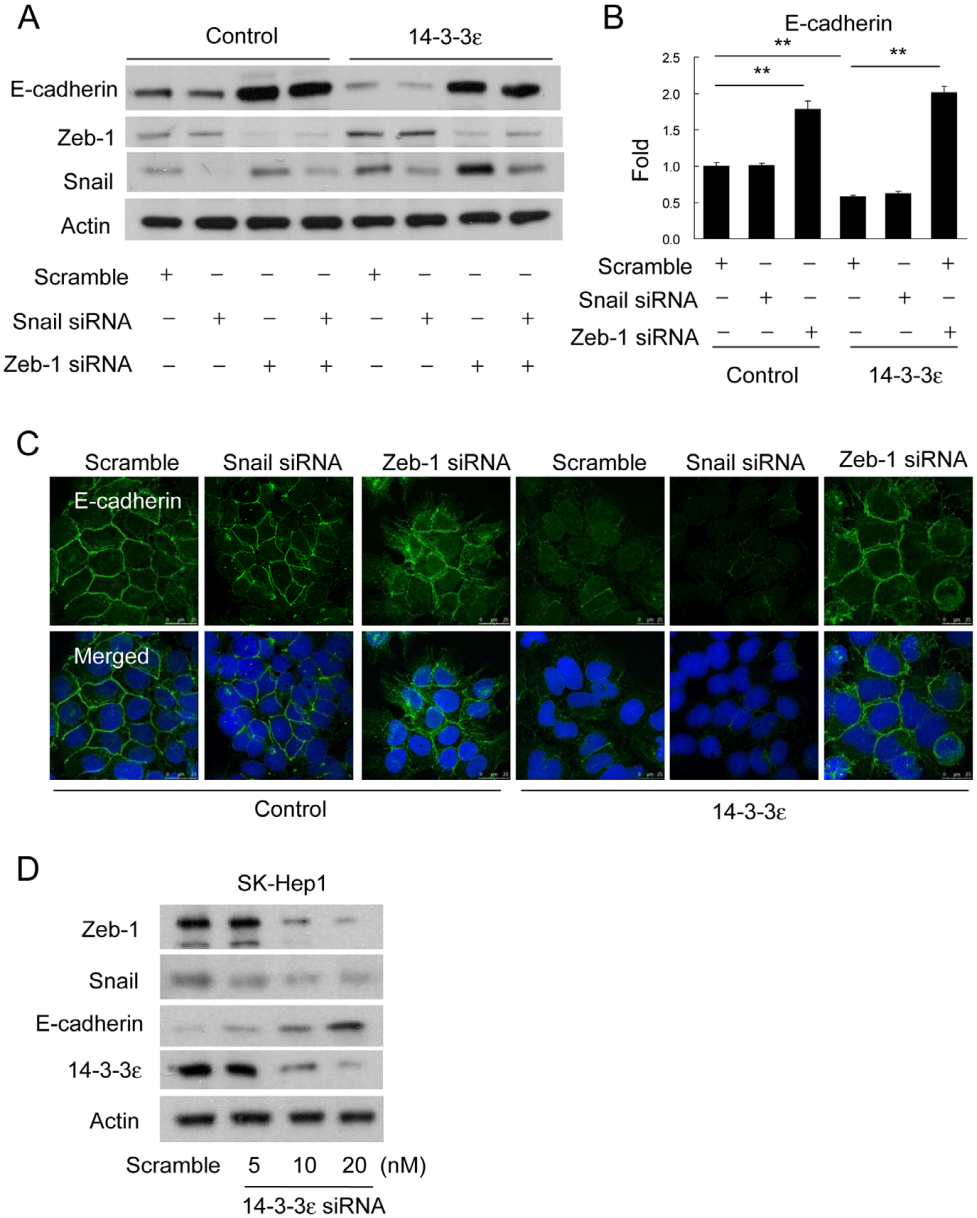
14-3-3ε suppresses E-cadherin expression *via* regulating Zeb-1. (A) Cells were transfected with scramble, Snail or/and Zeb-1 siRNAs for 48 hours. E-cadherin, Zeb-1, and Snail protein levels were determined by Western blotting analysis. Actin was used as loading control. (B) E-cadherin expression was determined by quantitative real-time PCR analysis in control and 14-3-3ε overexpression cells. These data are from three independent experiments and presented as the mean ± SD. **P<0.01. (C) Expression level and subcellular localization of E-cadherin was examined by immunofluorescent confocal microscopy. (D) 14-3-3ε siRNA dose-dependently decreased Zeb-1/Snail and restore E-cadherin expression in SK-Hep1 cells. Actin was used as loading control.

To further explore the role of Zeb-1 and Snail on 14-3-3ε-induced cell migration or EMT, we knockdown Zeb-1 and Snail with siRNAs and examined E-cadherin expression by Western blotting analysis. Interestingly, 14-3-3ε-reduced E-cadherin expression was specifically restored by Zeb-1 siRNA, but not by Snail siRNA (Figure 4A). This specific effect of Zeb-1 on regulating 14-3-3ε-reduced E-cadherin was validated by quantitative real-time PCR (Figure 4B). In addition, the selective effect of Zeb-1 knockdown restoring E-cadherin expression in 14-3-3ε overexpression cells was further confirmed by confocal microscopy (Figure 4C). SK-Hep1 cells expressed higher levels of 14-3-3ε and lower levels of E-cadherin than other HCC cell lines. We next transfected SK-Hep1 cells with 14-3-3ε siRNA and determined the expression of Zeb-1, Snail and E-cadherin by Western blotting analysis. Knockdown of 14-3-3ε reduced the expression of Zeb-1/Snail and induced that of E-cadherin in SK-Hep1 cells with a concentration-dependent manner (Figure 4D). These results demonstrate that the reduction of E-cadherin expression by 14-3-3ε is selectively mediated by regulation of Zeb-1. Thus, Zeb-1/E-cadherin expression is a downstream factor of 14-3-3ε for promoting EMT in HCC.

### Correlation and Impact of Positive 14-3-3ε with Negative E-cadherin Expression in HCC

**Figure 5:**
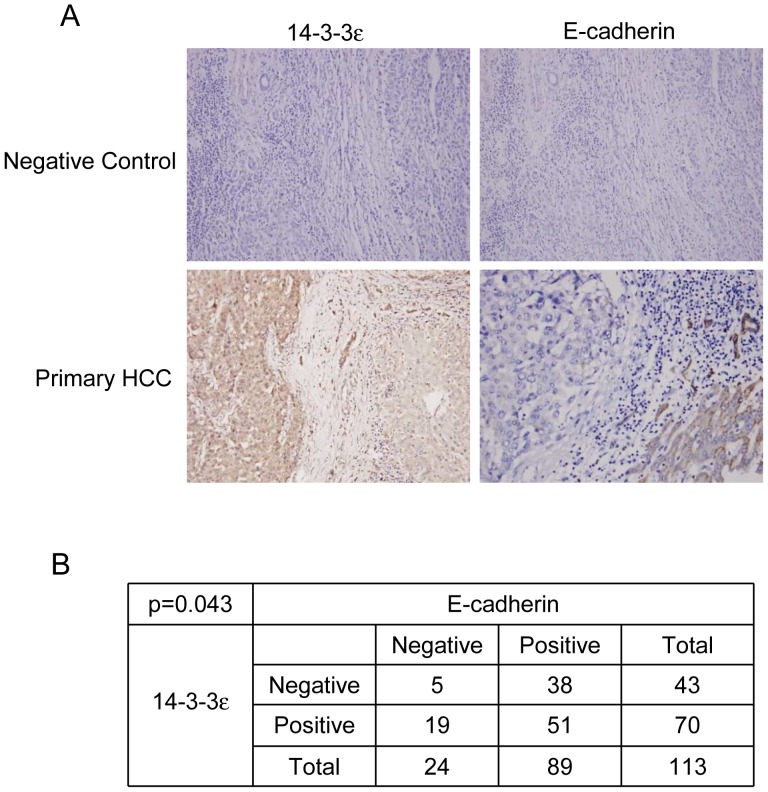
Reversed correlation of 14-3-3ε with E-cadherin expression of hepatocellular carcinoma tumors. (A) Representative expression of 14-3-3ε and E-cadherin in primary tissue of HCC examined by immunohistochemical analysis. Original magnification, ×200. (B) Significantly reversed correlation of 14-3-3ε with E-cadherin expression in primary HCC tumors was analyzed by Chi-square test.

To further support the likelihood that 14-3-3ε suppresses E-cadherin and regulates EMT as well as tumor progression, we examined the expression of 14-3-3ε and E-cadherin by immunohistochemical analysis in HCC tumors. Expression of 14-3-3ε was higher in HCC primary tumors than in the surrounding non-cancerous liver tissues (Figure 5A, left panel). We next determined the expression of E-cadherin and found that E-cadherin expression was reduced in HCC tumors (Figure 5A, right panel). Positive 14-3-3ε expression was significantly correlated with negative E-cadherin in HCC tumors (*p* = 0.043) (Figure 5B). In addition, expression of 14-3-3ε was correlated with Zeb-1 in HCC tumors (Figure S1).

### Association of 14-3-3ε/E-cadherin Expression with Extrahepatic Metastasis and Patient Survival of HCC

**Figure 6:**
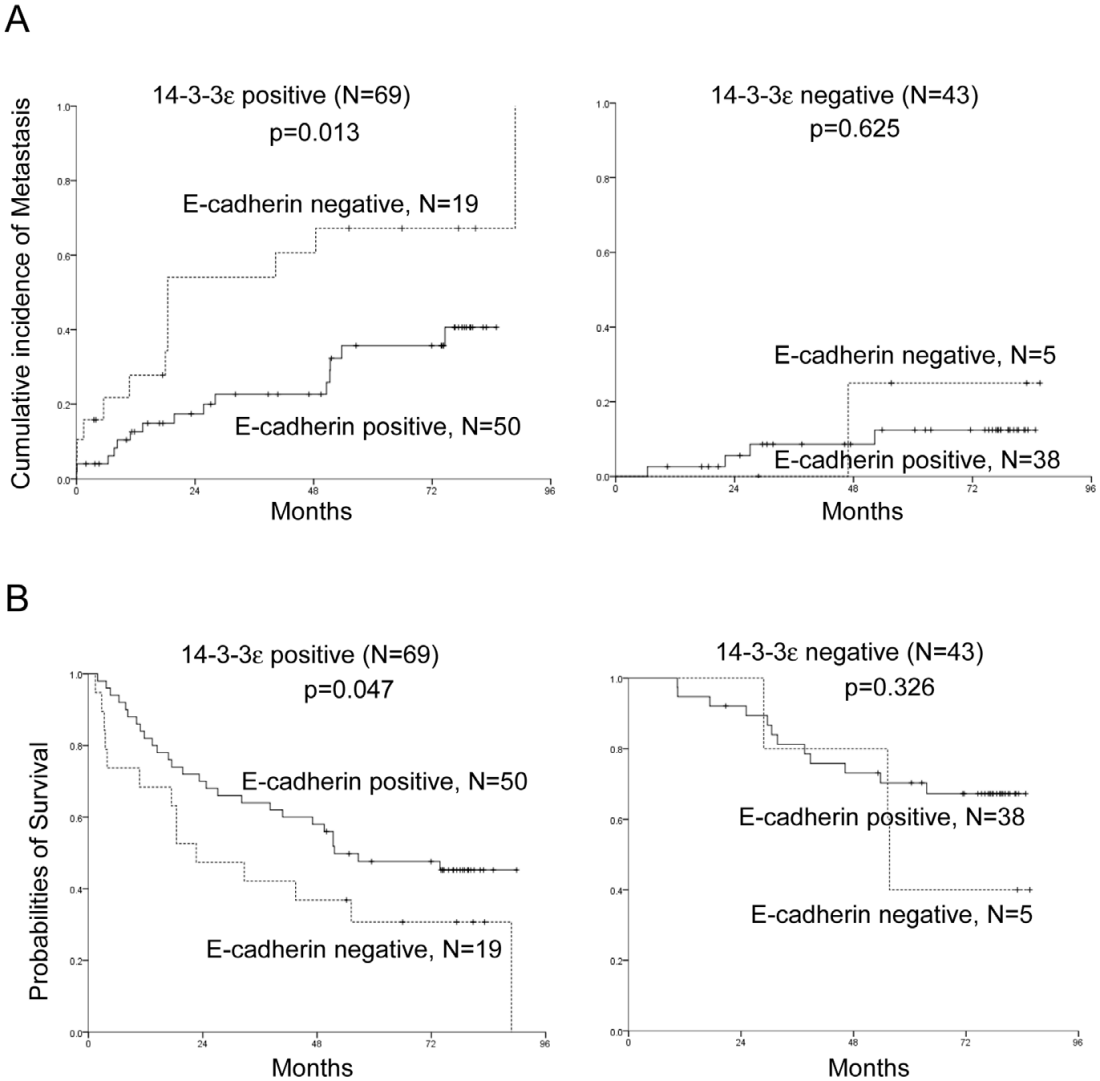
Kaplan-Meier analysis of 14-3-3ε and E-cadherin expression with prognostic outcomes in primary HCC tumors. E-cadherin positive expression reveals a (A) lower metastatic risk (*P* = 0.047), and (B) better overall survival rate (*P* = 0.013) when compared with E-cadherin negative in 14-3-3 positive patients.

We have previously shown that 14-3-3ε overexpression in HCC primary tumors was significantly associated with subsequent extrahepatic metastasis and reduced 5-year overall survival [30]. To evaluate whether E-cadherin plays an important role as a downstream effector of 14-3-3ε in promoting tumor progression, the associations of E-cadherin with clinicopathological characteristics and with 14-3-3ε expression were compared. In addition to 14-3-3ε positivity, expression of E-cadherin is significantly correlated with gender (*p* = 0.011), histology grade (*p* = 0.001), BCLC staging (*p* = 0.030), tumor size (*p* = 0.003), and subsequent extrahepatic metastasis (*p* = 0.004) (Table S3). Patients with positive E-cadherin expression exhibit a lower risk of metastasis (Figure 6A, *p* = 0.013) and better overall survival rate (Figure 6B, *p* = 0.047) than do those with negative E-cadherin expression in 14-3-3ε positive HCC tumors. These results provide clinical evidence to support the hypothesis that E-cadherin is one of the crucial downstream regulators of 14-3-3ε that modulate HCC tumor progression.

## Discussion

We previously demonstrated that 14-3-3ε expression is increased in primary and metastatic HCC. Elevated 14-3-3ε expression is correlated with higher risk of extrahepatic metastasis and lower survival rates of HCC patients [30]. In this study, we investigated the molecular mechanism to determine how 14-3-3ε regulates tumor progression. Attenuated expression of E-cadherin has been recognized as an important determinant and biomarker of tumor progression, one especially indicative of EMT in various tumors. In addition, gene silencing and loss of E-cadherin expression in the malignant progression of HCC have been demonstrated [6], [7], and it is suggested that E-cadherin is associated with reduced survival of HCC patients [8]. Our current investigation indicates that 14-3-3ε promotes HCC EMT and cell migration and also suppresses E-cadherin expression *via* upregulation of Zeb-1. We found that the expression of Zeb-1 was increased (14 of 113) in HCC primary tissues (Figure S1), although the increase is not as significant as in a previous report [14]. This difference may due to the sensitivity of reagents, sample size or differences in the cohort. It has been suggested that TGF-β and downstream signals of Smad2/3 activation regulate Zeb expression and EMT [41]. Although we performed the experiments of Smad2 knockdown in 14-3-3ε overexpression cells, we did not observe significant restoration of E-cadherin (Figure S2). In addition, increased 14-3-3ζ expression has been shown to promote EMT *via* associating with TGF-β receptor signaling and PI3-K subunit p85 in breast cancer cells [42], [43]. However, treating cells with TGF-β receptor or PI3-K inhibitors (SB-431542 or LY-294002) did not abolish E-cadherin-suppression induced by 14-3-3ε (Figure S3). These results suggest the effect of 14-3-3ε-suppressed E-cadherin expression may not be regulated through TGF-β/Smad2/3 or PI3-K signal pathways. Thus, 14-3-3ε contributes to EMT via induction of Zeb-1 may be mediated by a novel mechanism. Further work is currently ongoing to investigate how 14-3-3ε regulates Zeb-1 expression.

A functional motif for 14-3-3 binding in Snail was demonstrated, and the ternary protein complex comprised of 14-3-3, Ajuba and Snail plays an important role in transcriptional repression and EMT [44]. Snail contains two potential phosphorylated residues at Ser11 and Thr177 in putative motifs for 14-3-3 protein binding [44]. Phosphorylated Snail selectively interacts with 14-3-3γ, 14-3-3ε, 14-3-3θ/τ, 14-3-3η and 14-3-3β, but not with isoforms of 14-3-3σ and 14-3-3ζ [44]. This interaction has been demonstrated to be required for E-cadherin suppression in MCF breast cancer cells and 293 cells [44]. To find out whether 14-3-3ε suppresses E-cadherin *via* a similar mechanism, we co-immunoprecipitated 14-3-3ε stable cells with anti-Flag antibody, followed by Western blotting analysis of Zeb-1. However, no significant interaction of 14-3-3ε with Zeb-1 was detected (Figure S4). Further studies using proteomic approaches are currently underway to investigate the potential 14-3-3ε interaction partners in regulating Zeb-1 and EMT of HCC.

Expression of transcriptional repressors for E-cadherin, including Zeb-1, Snail, SIP1 and Twist, is associated with cell invasion, EMT, metastasis and poor patient survival of HCC [12]-[16]. However, no previous studies have shown that 14-3-3ε promotes HCC tumor progression *via* modulating E-cadherin transcriptional repressors. Our study shows for the first time that 14-3-3ε induces Zeb-1 expression, thereby repressing E-cadherin expression and promoting EMT. The 14-3-3ε regulation of E-cadherin reduction occurs through Zeb-1, and not through Snail or other E-cadherin repressors, as supported by Figure 3A. To further clarify the regulation of 14-3-3ε-reduced E-cadherin expression by Zeb-1, 14-3-3ε overexpression cells were transfected with Zeb-1 siRNA or control scramble siRNA, and the gene expression profile was analyzed by use of microarray analysis. Altered gene expression (fold change >2) was identified of 557 transcripts in 14-3-3ε overexpression *vs.* the control cells and 160 transcripts in Zeb-1 siRNA *vs.* scramble siRNA cells. Among them, *CDH1* (E-cadherin), *SMAD2*, and *PLA2G2A* were regulated in 14-3-3ε overexpression cells but had a reversed expression pattern in Zeb-1 knockdown cells (Data not shown). These results provide additional evidence to support our findings.

In addition to Zeb-1, our results indicated that 14-3-3ε induces Snail expression and promotes HCC cell migration (Figure 3A and 3E). However, knockdown of Snail did not restore 14-3-3ε-reduced E-cadherin expression (Figure 4A and 4B). Interestingly, partially increased of Snail expression was found by treatment with Zeb-1 siRNA (Figure 4A). As Snail and Zeb-1 regulate EMT of HCC may be mediated by separate and complicated pathways, a compensative effect is possibly involved. Further investigation is needed to elucidate this finding. Additionally, our results indicated that 14-3-3ε overexpression-induced EMT (increase of N-cadherin, Vimentin, Zeb-1 and Snail as well as decrease of E-cadherin expression) was impaired by 14-3-3ε siRNA (Figure 2C and Figure 3D). However, knockdown of 14-3-3ε has no significant effect on affecting EMT markers in control cells (Figure 2C and Figure 3D). We therefore postulate that other endogenous house-keeping regulators may be involved in maintaining basal level of Snail/Zeb-1 expression. Endogenous level of Snail/Zeb-1 modulates expression of EMT markers which is independent of 14-3-3ε expression in HCC. Moreover, 14-3-3ε may upregulate FAK expression *via* activation of NFκB to enhance HCC cell migration [35]. These results reveal the complicated signal mechanisms that are involved in 14-3-3ε induced HCC cell migration, EMT, and metastasis. Uncovering the complex role of 14-3-3ε in tumor progression could contribute to the development of therapeutic strategies for treatment of aggressive and advanced HCC.

## Supporting Information

Figure S1 Representative immunohistochemical analysis of 14-3-3ε and Zeb-1 in HCC tissues. 14-3-3ε and Zeb-1 are positive expressed in HCC tumors.
(TIF)

Figure S2 **Smad-2 does not regulate 14-3-3ε/E-cadherin expression.** Smad-2 siRNA has no significant effect on the restoration of 14-3-3ε-reduced E-cadherin expression.
(TIF)

Figure S3 **TGF-β and PI3K/Akt do not regulate 14-3-3ε/E-cadherin expression.** Treatment with SB431542 (TGF-βR Inhibitor) or LY294002 (PI3K Inhibitor) has no significant effect on the restoration of 14-3-3ε-reduced E-cadherin expression.
(TIF)

Figure S4 **14-3-3ε does not direct interact with Zeb-1.** No significant interaction between 14-3-3ε and Zeb-1 was observed by using co-immunoprecipitation. Cell lysates were collected and subjected to Protein A magnet beads (Millipore) immunoprecipitation followed by Western blot analysis.
(TIF)

Table S1 **Oligonucleotide sequences of small interfering RNAs.** siRNA sequences for *YWHAE* (14-3-3ε), *SNAI1* (Snail) and *ZEB1* (Zeb-1).
(TIF)

Table S2 **Oligonucleotide sequences for Q-PCR procedures.** Primer sequences for *CDH1* (E-cadherin), *SNAI1* (Snail), *ZEB1* (Zeb-1), *SMAD2* (Smad-2) and *PLA2G2A*.
(TIF)

Table S3 **Correlation of E-cadherin expression with 14-3-3ε and clinicopathological characteristics in primary HCC patients.** One-way ANOVA was used to analyze correlation between clinicopathological parameters and 14-3-3ε with E-cadherin expression.
(TIF)
