# Supplementary figures and images for: 14-3-3ε Overexpression Contributes to Epithelial-Mesenchymal Transition of Hepatocellular Carcinoma
Source: PLoS One. 2013 Mar 6;8(3):e57968. doi: 10.1371/journal.pone.0057968 (PMC3590290; doi:10.1371/journal.pone.0057968)

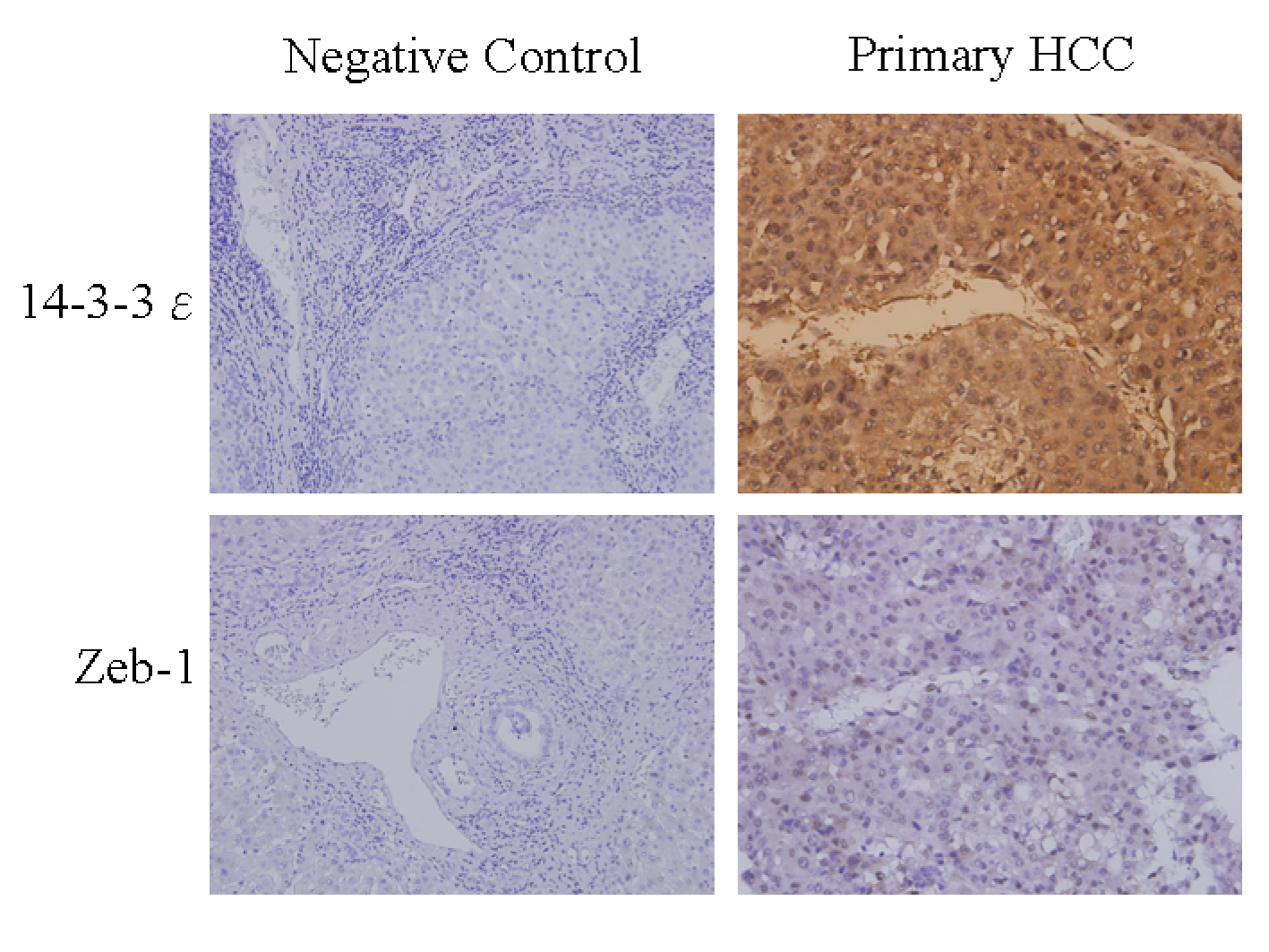

Supplement: Figure S1 — Representative immunohistochemical analysis of 14-3-3ε and Zeb-1 in HCC tissues. 14-3-3ε and Zeb-1 are positive expressed in HCC tumors. (TIF) [file pone.0057968.s001.tif]

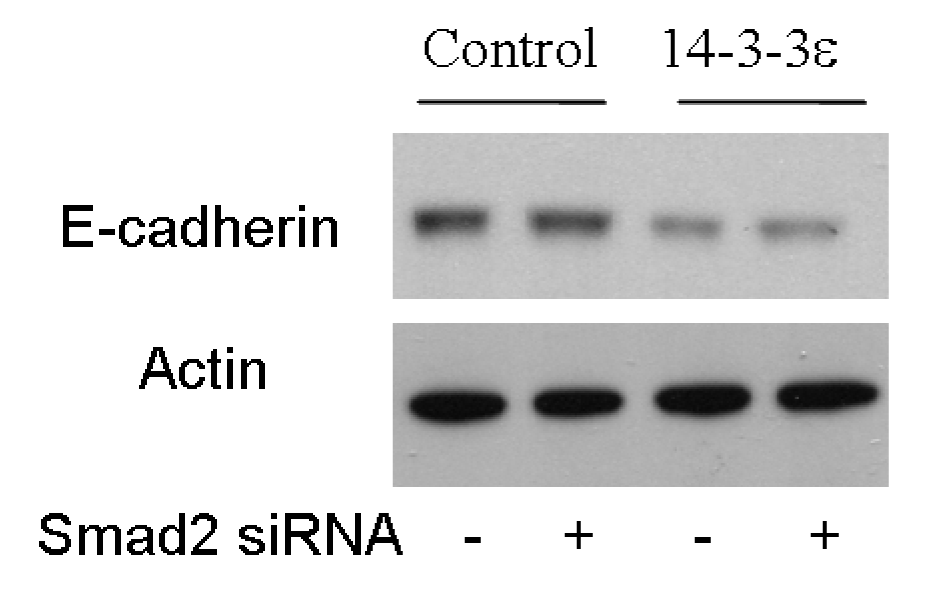

Supplement: Figure S2 — Smad-2 does not regulate 14-3-3ε/E-cadherin expression. Smad-2 siRNA has no significant effect on the restoration of 14-3-3ε-reduced E-cadherin expression. (TIF) [file pone.0057968.s002.tif]

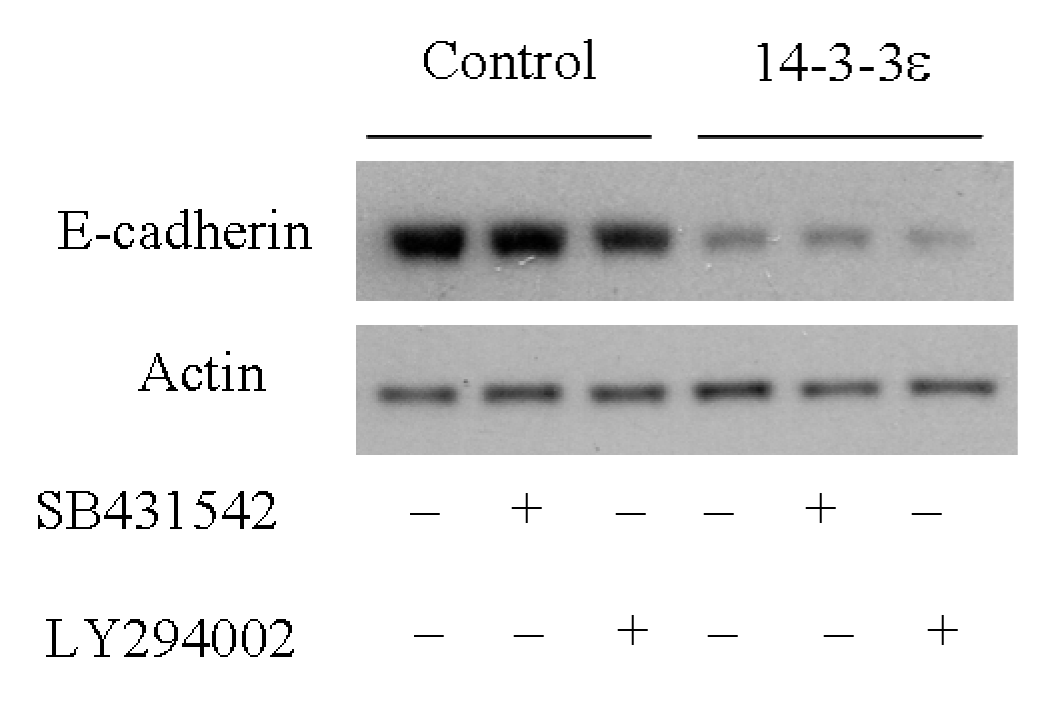

Supplement: Figure S3 — TGF-β and PI3K/Akt do not regulate 14-3-3ε/E-cadherin expression. Treatment with SB431542 (TGF-βR Inhibitor) or LY294002 (PI3K Inhibitor) has no significant effect on the restoration of 14-3-3ε-reduced E-cadherin expression. (TIF) [file pone.0057968.s003.tif]

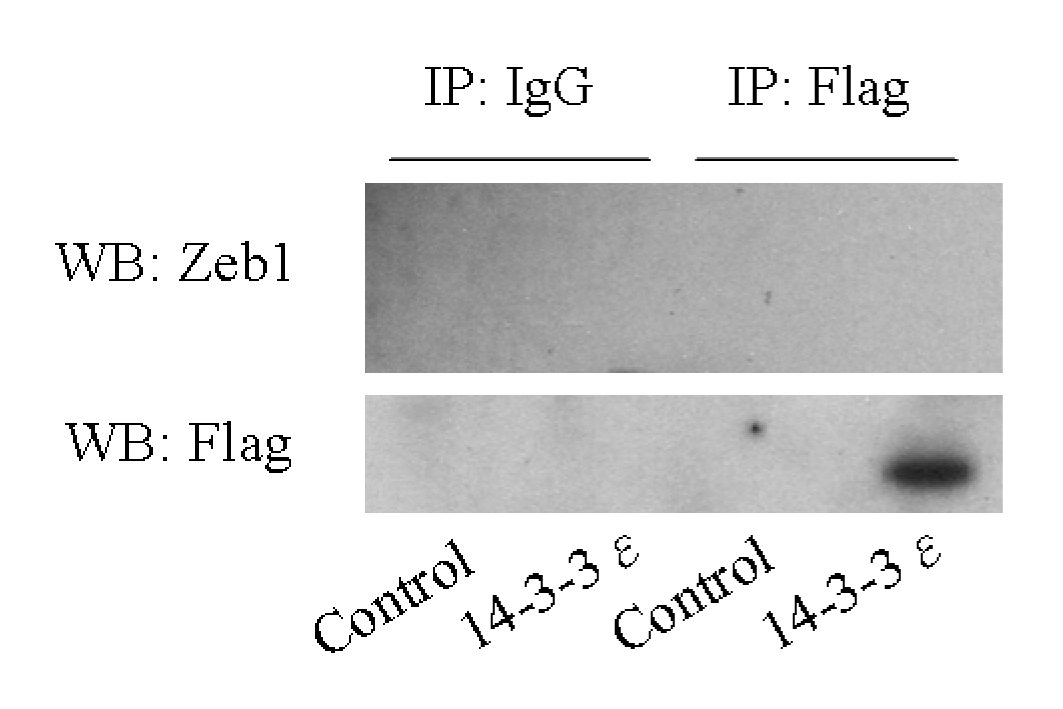

Supplement: Figure S4 — 14-3-3ε does not direct interact with Zeb-1. No significant interaction between 14-3-3ε and Zeb-1 was observed by using co-immunoprecipitation. Cell lysates were collected and subjected to Protein A magnet beads (Millipore) immunoprecipitation followed by Western blot analysis. (TIF) [file pone.0057968.s004.tif]

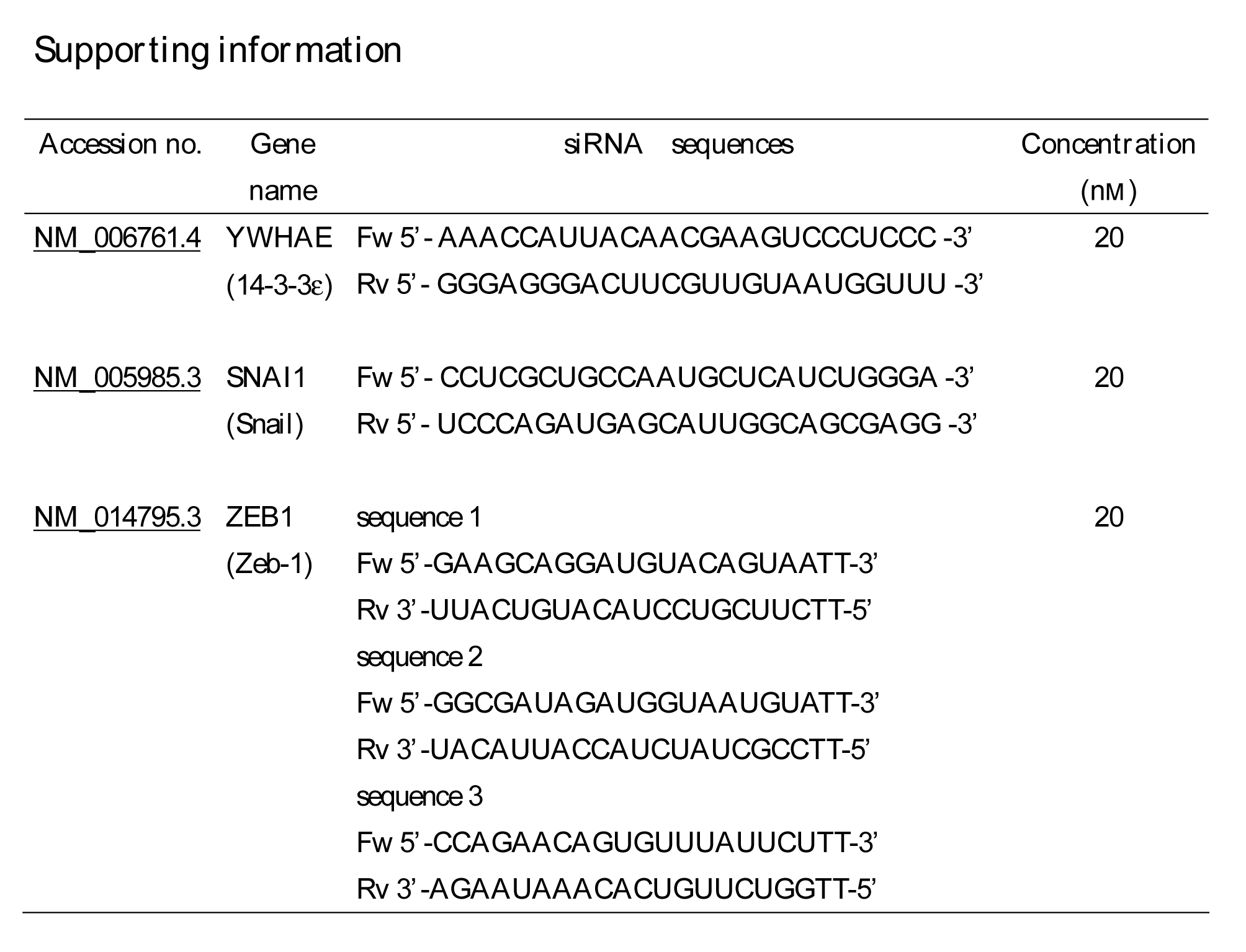

Supplement: Table S1 — Oligonucleotide sequences of small interfering RNAs. siRNA sequences for YWHAE (14-3-3ε), SNAI1 (Snail) and ZEB1 (Zeb-1). (TIF) [file pone.0057968.s005.tif]

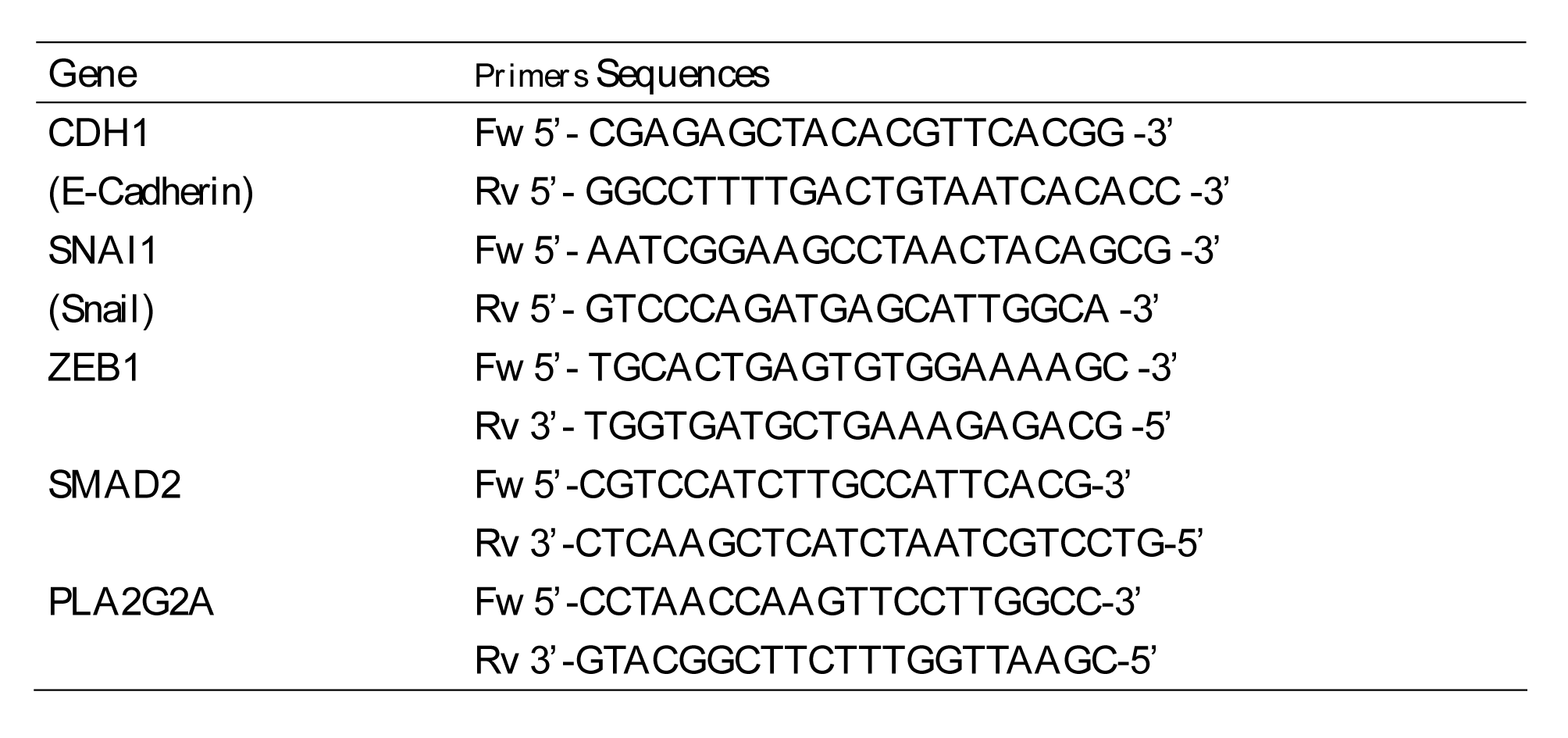

Supplement: Table S2 — Oligonucleotide sequences for Q-PCR procedures. Primer sequences for CDH1 (E-cadherin), SNAI1 (Snail), ZEB1 (Zeb-1), SMAD2 (Smad-2) and PLA2G2A. (TIF) [file pone.0057968.s006.tif]

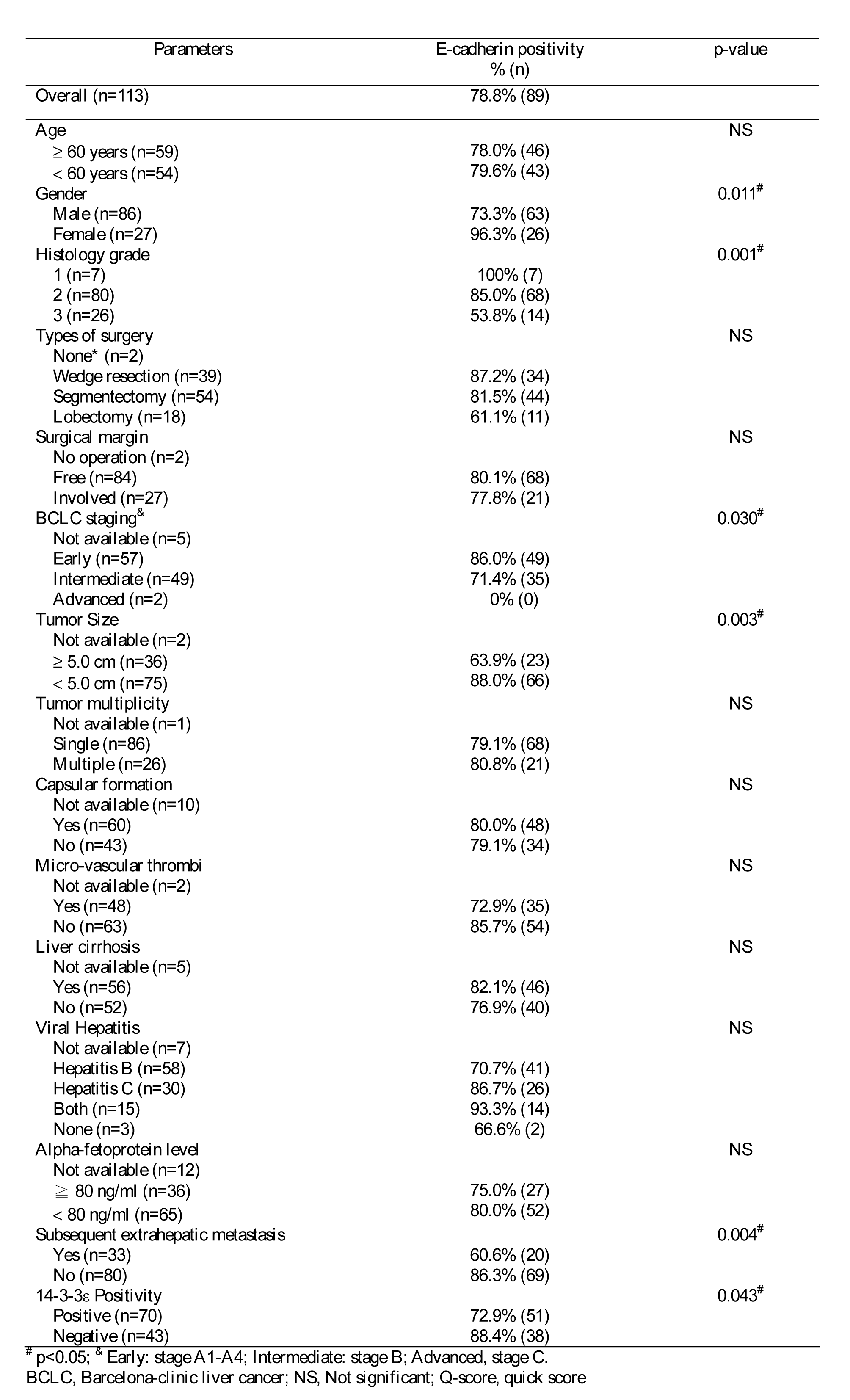

Supplement: Table S3 — Correlation of E-cadherin expression with 14-3-3ε and clinicopathological characteristics in primary HCC patients. One-way ANOVA was used to analyze correlation between clinicopathological parameters and 14-3-3ε with E-cadherin expression. (TIF) [file pone.0057968.s007.tif]
